# Surgical Management of Epidermoid Cysts of Scalp: A Case Report

**DOI:** 10.7759/cureus.71891

**Published:** 2024-10-19

**Authors:** Yajas Kumar, Rakshak Anand, Nitin Bhagat, Kapila Chakarvarty, Yashmi Jaiswal

**Affiliations:** 1 Department of Oral and Maxillofacial Surgery, Manav Rachna Dental College, Faridabad, IND; 2 Oral and Maxillofacial Surgery, Private Practice, New Delhi, IND

**Keywords:** cyst, epidermoid cysts, oral and maxillofacial surgery, scalp lump, surgical removal

## Abstract

Epidermoid cysts represent a common benign cutaneous neoplasm resulting from the invagination and subsequent proliferation of epidermal cells within the dermal layer. They exhibit a predilection for the face, cervical region, upper torso, and scalp, manifesting as firm, mobile nodules with a characteristic central punctum. Surgical excision is the recommended therapeutic approach for sizeable or recurrent cysts, particularly when they elicit discomfort or aesthetic concerns. Although recurrence is possible, especially in cases of incomplete excision, adherence to proper scalp hygiene and gentle grooming practices may mitigate the risk of cyst formation, particularly in the commonly affected scalp region. It is noteworthy that while these lesions are generally benign, proper diagnosis and management are crucial to exclude more serious pathologies and prevent potential complications. Here we present a case of epidermoid cysts on the scalp of a male patient managed with surgical excision.

## Introduction

An epidermoid cyst is a common, non-cancerous growth on the skin that arises from the proliferation of epidermal cells within the dermis. Epidermal cells, which form the outermost layer of the skin, sometimes get trapped beneath the surface due to factors such as trauma, inflammation, or developmental anomalies leading to the formation of epidermoid cysts. They are most often found on the face, neck, and upper body, and are characterized by a firm, movable nodule often with a visible central punctum. The scalp is a particularly susceptible area for the development of epidermoid cysts due to its high concentration of hair follicles and sebaceous glands [1]. These structures create an ideal environment for cyst formation, particularly when hair follicles become blocked or damaged, trapping keratin-producing epidermal cells beneath the surface. While it is not always possible to prevent these cysts, practicing good scalp hygiene and avoiding excessive friction while grooming can help reduce the risk. As a result, epidermoid cysts on the scalp are fairly common and can occasionally cause discomfort or cosmetic issues, especially if they grow in size or become inflamed [2].

## Case presentation

**Figure 1:**
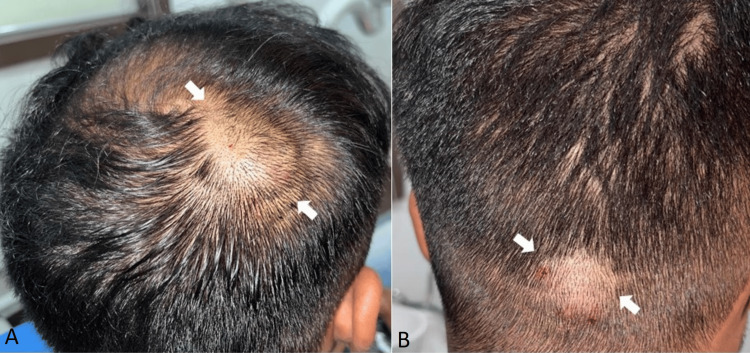
Well circumscribed painless mass present in the right posterior parietal triangle region (A) and in mid-occipital region (B)

A 29-year-old male reported to the outpatient department of Manav Rachna Dental College, Haryana with a chief complaint of swelling and discomfort with respect to the right and back side of his scalp. Pain occurred intermittently over two years with a marginal increase in size. The patient gave no history of trauma or any past surgical intervention. General physical examination was normal and no abnormalities were detected - it was done to rule out nevoid basal-cell carcinoma syndrome and Gardner's syndrome. On examination, a 2x2 cm painless mobile mass was palpated in the right posterior parietal triangle region, and a 2x3 cm mobile mass with respect to the mid-occipital region (Figure 1).

**Figure 2:**
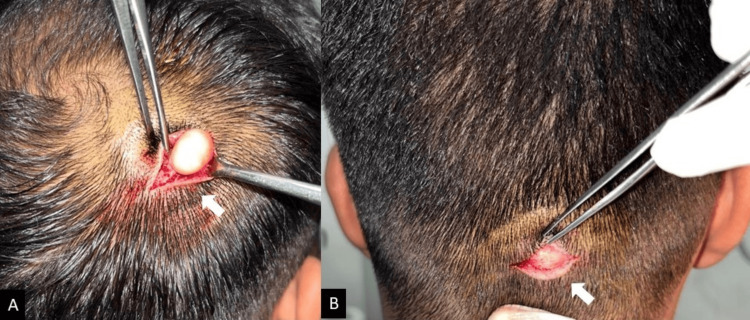
Isolation of cystic lesions from surrounding tissues after sharp dissection was done in right posterior parietal region (A) and mid-occipital region (B).

The skin overlying appeared to be normal with no alteration in the hair morphology. Based on the findings, a provisional diagnosis of epidermoid cyst was made. A plan to excise the lesion under local anesthesia was made after due consent from the patient. Under all aseptic conditions, the hair surrounding the swellings was trimmed with a surgical clipper, followed by local infiltration using 2% lidocaine hydrochloride with adrenaline (1:80,000). An elliptical incision was made around the lesions using a No. 15 blade (Bard-Parker® Stainless Steel, Aspen Surgical, Caledonia, UA) followed by a sharp dissection separating it from the surrounding tissues (Figure 2).

**Figure 3:**
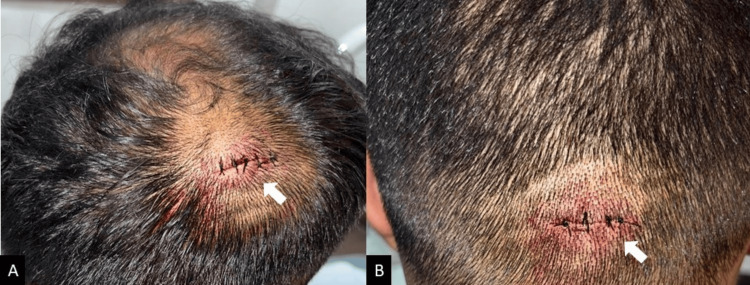
Primary closure was achieved via simple interrupted sutures using 3/0 silk on a reverse cutting needle in both operative sites (A) and (B).

Electrocautery was kept on standby in case of any small bleeders. The cysts were excised successfully, followed by irrigation and tight closure using 3/0 silk and a pressure dressing to prevent hematoma formation (Figure 3).

Antibiotic therapy was initiated for 10 days to prevent any staphylococcal infection. Histopathological analysis confirmed the provisional diagnosis revealing a cystic lesion lined by para-keratinized stratified squamous epithelium of 2-3 cell layer thickness with flattened cells and well-developed granular cell layer and no skin appendages. The connective tissue wall showed dense bundles of collagen fibers with areas of hyalinization and few blood vessels. The wounds healed uneventfully and the patient is on periodic follow-up for two years without any sign of recurrence.

## Discussion

An epidermoid cyst, also referred to as an epidermal inclusion cyst, is a benign lesion resulting from the abnormal accumulation of keratinous material within a cystic structure lined by epidermal cells. The pathophysiology of this condition involves the displacement or entrapment of epidermal elements beneath the skin's surface. This process may be triggered by factors such as trauma, surgery, inflammation, or congenital anomalies, all of which contribute to the entrapment of keratin-producing epidermal cells within the dermis or subcutaneous tissue.

Epidermoid cysts are more commonly observed in individuals with a history of active or previous acne, likely due to the altered dynamics of sebaceous glands and hair follicles associated with the condition. Approximately 80% of these cysts present as solitary, painless nodules, remaining benign and causing minimal discomfort. However, in around 20% of cases, they become painful, primarily due to secondary bacterial infections that provoke an inflammatory response. Typically, these cysts are freely movable beneath the skin, reflecting their non-adherent nature. Nevertheless, recurrent infections may lead to fibrosis, resulting in the cysts becoming fixed or less mobile due to the formation of surrounding scar tissue. This fibrosis is essentially the body’s protective mechanism, encasing the cyst in a layer of fibrous tissue following repeated inflammation or infection.

From a pathophysiological perspective, the infundibular epithelium, which lines the upper part of the hair follicle, often proliferates in response to inflammation or irritation. This hyperproliferation leads to the trapping of keratin within the cyst, causing its characteristic swelling and growth. Moreover, the presence of multiple epidermoid cysts is frequently associated with hereditary conditions such as Gardner syndrome, a genetic disorder characterized by intestinal polyps and other neoplasms, and Gorlin syndrome, which is marked by a predisposition to basal cell carcinomas, jaw cysts, and skeletal abnormalities. In such cases, the appearance of these cysts may serve as a clinical clue to a more intricate systemic condition, requiring further diagnostic exploration and a multidisciplinary approach to management. syndrome [3].

The cyst is encapsulated by a wall that mimics the normal epidermis, composed of stratified squamous epithelium, which continues to proliferate and produce keratin, much like normal skin. However, in the context of the cyst, this keratin has no way to be shed as it would on the skin surface. Instead, it accumulates within the cystic structure, forming a semi-solid, often malodorous material that can cause gradual enlargement of the cyst. The keratinized debris is characteristically "cheesy" in consistency, a hallmark of epidermoid cysts [4]. On a cellular level, the pathophysiology of epidermoid cyst formation is linked to the dysregulation of keratinocyte proliferation and differentiation. In normal epidermis, basal keratinocytes undergo differentiation, migrating towards the surface where they form the stratum corneum and eventually slough off. However, in the case of an epidermoid cyst, this cycle is disrupted. Keratinocytes become trapped in the dermis or deeper layers, and the continuous keratin production leads to an expanding cystic mass [5]. When the cyst remains intact, it is usually asymptomatic and slowly enlarging. However, if the cyst ruptures, the keratin contents spill into the surrounding tissue, leading to an acute inflammatory response. This response is mediated by the immune system's recognition of keratin as a foreign substance in the dermis, triggering the infiltration of neutrophils and macrophages. These immune cells attempt to clear the keratin, but the inflammatory cascade can result in pain, swelling, and redness. In severe cases, an epidermoid cyst may become secondarily infected, which further exacerbates inflammation and can lead to abscess formation [6,7].

Clinically, epidermoid cysts present as slow-growing, round, and firm subcutaneous nodules, often with a visible punctum, which represents the blocked hair follicle from which the cyst originated. While generally painless, they can become tender and swollen if infected or inflamed [8]. Rarely, large or recurrent cysts can lead to significant cosmetic or functional concerns, especially if they are located on visible or sensitive areas such as the face, neck, or scalp. In very rare cases, there have been reports of squamous cell carcinoma arising from long-standing or recurrent epidermoid cysts, underscoring the need for careful monitoring of atypical or rapidly enlarging lesions [9].

The management of an epidermoid cyst entails a judicious combination of clinical acumen and precise surgical intervention. In asymptomatic cases, the lesion may be observed conservatively, particularly if it poses no functional impairment or aesthetic concern. However, when intervention is warranted; be it due to inflammation, infection, or patient preference-the therapeutic approach must be meticulously tailored to ensure complete eradication while minimizing recurrence. The gold standard for definitive treatment remains the excision of the cyst in toto, including the cyst wall. The surgical technique demands an expert hand to ensure the entire cyst capsule is removed intact, as incomplete excision may result in recurrence [10]. This procedure is often performed under local anesthesia, ensuring patient comfort while allowing for a precise dissection. In certain cases, a minimal excision technique is employed, wherein a small incision is made, and the cyst is drained before the sac is carefully extracted. This method offers a less invasive option with superior cosmetic outcomes, albeit with a slight risk of recurrence.

In instances of infected or inflamed cysts, incision, and drainage may be required initially to alleviate acute symptoms. Antibiotic therapy is frequently employed to manage secondary bacterial infections; however, it does not resolve the cyst itself [11]. Once the infection has subsided, delayed excision is recommended to prevent the development of a residual abscess or chronic inflammation. In patients who prioritize cosmetic outcomes, particularly when the cyst is located in cosmetically sensitive regions such as the scalp or face, the application of fine surgical techniques, including punch excision or elliptical excision, may be considered to minimize scarring. Postoperative care involves careful wound management to support optimal healing, potentially supplemented by silicone gel sheets or other scar reduction therapies [12].

## Conclusions

The pathophysiology of epidermoid cysts involves the aberrant entrapment of keratinocytes, leading to continuous keratin production within a cystic structure. Over time, this results in the formation of a slowly expanding nodule filled with keratinous debris. While usually benign and asymptomatic, complications such as rupture, infection, or inflammation can occur, leading to more pronounced clinical symptoms. The treatment of epidermoid cysts demands a discerning clinical judgment, coupled with the finesse of surgical intervention, to achieve an optimal outcome, balancing both functional and aesthetic considerations.
